# Cancer risk in patients with diabetic nephropathy: A retrospective cohort study in Hong Kong

**DOI:** 10.1097/MD.0000000000008077

**Published:** 2017-09-22

**Authors:** Chi Yuen Cheung, Maggie Kam Man Ma, Wai Leung Chak, Sydney Chi Wai Tang

**Affiliations:** a Department of Medicine, Renal Unit, Queen Elizabeth Hospital, Hong Kong SAR; b Department of Medicine, Division of Nephrology, The University of Hong Kong, Queen Mary Hospital, Hong Kong SAR.

**Keywords:** albuminuria, cancer, diabetes mellitus, nephropathy

## Abstract

Diabetic nephropathy (DN) is a leading cause of end-stage kidney disease nowadays. Certain cancers are more common in patients with diabetes mellitus. However, there are no data concerning the cancer pattern in patients with DN. The aim of this study is to investigate the site-specific cancer risk and mortality in these patients.

A retrospective cohort study of 5643 DN patients between 2000 and 2015 was conducted in 2 large hospitals in Hong Kong. Incidence and mortality of various cancers were compared with those of general population using standardized incidence ratios (SIRs) and standardized mortality ratios (SMRs) respectively.

With 24,726 person-years follow-up, 250 cancers were diagnosed. Overall cancer incidence was similar between DN patients and the general population (SIR 1.05, 95% confidence interval [CI] 0.92–1.19). However, certain site-specific cancers are increased in DN patients: the highest risk was observed for laryngeal cancer (SIR 3.03, 95% CI 1.11–6.60), followed by cancers of liver (SIR 1.96, 95% CI 1.35–2.76) and colorectum (SIR 1.92, 95% CI 1.53–2.37), but the risk of prostate cancer was lower (SIR 0.48, 95% CI 0.21–0.95) in the males with DN. The SMR of all cancers was 1.17 (95% CI 1.01–1.37). For individual specific site, only colorectal cancer carried a significant higher mortality risk (SMR 2.45, 95% CI 1.82–3.23).

Our data suggested that DN is associated with increased incidence of cancers of colorectum, liver, and larynx but decreased incidence of prostate cancer. Moreover, there is increased mortality of colorectal cancer in patients with DN.

## 1 Introduction

The prevalence of diabetes mellitus (DM) is increasing worldwide with an aging population and lifestyle changes in many countries. DM represents a major challenge in public health because it is associated with many acute and chronic complications which seriously affect the quality of life and survival of affected individuals. The association between DM and cancer has been studied extensively in the past and most of the studies showed that certain types of cancer are more common in patients with type 1 or type 2 DM.^[1–8]^ In addition, DM has also been shown to be associated with cancer mortality.^[8,9]^

Albuminuria is an early sign of diabetic nephropathy (DN), which occurs in 25% to 40% of patients with DM.^[10,11]^ DN is now the leading cause of end-stage kidney disease (ESKD) in different countries.^[12]^ Various studies found that urinary albumin excretion alone can predict cancer mortality even in people without diabetes.^[13,14]^ In addition, albuminuria has been observed in patients with different types of cancers such as colorectal, lung, renal cell, breast, and non-Hodgkin lymphoma.^[15–19]^

Despite all these evidence, there are no data concerning the cancer pattern in patients suffering from DN. The aim of our study is to investigate the risk and mortality of various cancers in patients suffering from DM associated nephropathy.

## 2 Methods

This is a retrospective cohort study in all patients with DN, who had follow-up in the outpatient clinics of the Queen Elizabeth Hospital and Queen Mary Hospital, 2 of the largest hospitals in Hong Kong, between the period January 1, 2000 and December 31, 2015. Those patients with DN were identified from the Clinical Data Analysis and Reporting System (CDARS).

The CDARS is a tool developed by the Hospital Authority, which is a statutory body responsible for primary, secondary, and tertiary public healthcare services of Hong Kong. The database generated from CDARS contains patients’ demographic data and clinical information including diagnoses, operations, drug prescriptions, laboratory investigations, inpatient stays, and outpatient clinics since 1993. In addition, it also contains data on causes of death through its internal linkage to regional death registry from the Immigration Department.

The inclusion criteria included reported primary or secondary diagnosis of DM or requirement of medication for the treatment of DM, together with the presence of macroalbuminuria or proteinuria during screening for the diabetic complications. Macroalbuminuria was determined by measuring the amount of albumin in a spot urine sample and was defined as the urinary albumin to creatinine ratio (ACR) >30 mg/mmol. However, proteinuria could be detected by either spot urinary protein to creatinine ratio (PCR) >0.5 g/g (56 mg/mmol) or 24-hour urinary protein >0.5 g.^[20,21]^ The exclusion criteria included missing values for determination of albuminuria or proteinuria; presence of kidney diseases other than DN, and history of organ transplants. The cohort entry date was defined as the date of DN when macroalbuminuria or proteinuria was first diagnosed. Basic demographic and clinical data including sex, age, diagnosis of DM and cancers, amount of albuminuria and/or proteinuria, hemoglobin (Hb) A1c, and serum creatinine level were extracted from the CDARS and individual patient's medical record. Estimated glomerular filtration rate was calculated by the abbreviated MDRD equation (mL/min/1.73 m^2^): 186 × (creatinine/88.4)−1.154 × (age)−0.203 × (0.742 if female) × (1.210 if black).^[22]^ All cancers were diagnosed with histology and other relevant information, such as radiological imaging, and coded according to the 10th World Health Organization *International Classification of Disease* (*ICD-10*). The follow-up duration was defined from the date of cohort entry to the date of death, the last reported contact, or March 31, 2016. This study was approved by the research ethics committee of both hospitals.

### 2.1 Statistical Analyses

The statistical analyses were performed by SPSS (SPSS 20.0, Inc, Chicago, IL). Categorical data were expressed as percentages and continuous data were expressed as mean ± standard deviation (SD) or median (range). Categorical data were compared with *χ*^2^ or Fisher exact tests, whereas continuous data were compared with *t* test or Mann-Whitney *U* test. The outcome in this study included the standardized incidence ratio (SIR) and the standardized mortality ratio (SMR) of different cancers in patients with DN. The SIR was calculated as the number of observed cancer cases per 100,000 patient-years among the patients with DN divided by the expected number of cancer cases per 100,000 patient-years. The expected number of cases was based on the age-adjusted cancer incidence rates in the general population. All information about cancer incidence and mortality in the Hong Kong general population could be obtained from the Hong Kong Cancer Registry 2011.^[23]^ The 95% confidence intervals of the SIRs were calculated by assuming that the observed cancers follow a Poisson distribution. For patients with multiple primary cancers, each tumor was counted separately in the analysis and the cancer would not be counted if it was diagnosed before the onset of DN. However, the SMR was the observed rate of cancer death among the patients with DN divided by the expected rates of cancer death and the 95% confidence intervals (CIs) were also calculated by assuming that the observed mortality follow a Poisson distribution. A *P* value of <.05 was defined as statistically significant.

## 3 Results

Total 6218patients were diagnosed to have DM with the presence of macroalbuminuria or proteinuria during the study period. Among them, 575 patients were excluded (425 with history of kidney or liver transplants and 150 because of the presence of other renal diseases). Thus, only 5643 patients were included in the analysis. The clinical characteristics of these patients are depicted in Table 1.

**Table 1:**
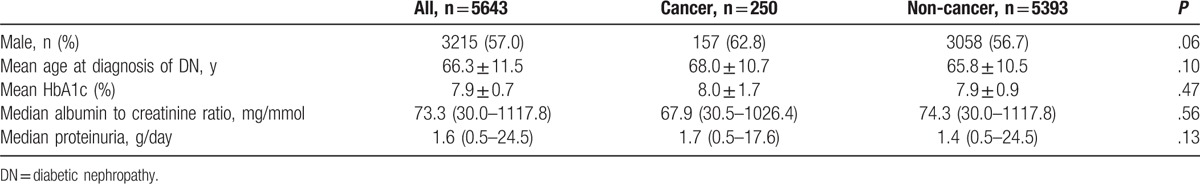
Clinical characteristics of DN patients analyzed in the study.

After a median follow -up duration of 4 years (6 months–16 years), 250 cancers were diagnosed. The overall cancer incidence rate was 4.4%. In the cancer group, the age at diagnosis of DN was 68.0 ± 10.7 years, whereas the age at diagnosis of cancer was 72.0 ± 9.7 years. There was no significant difference in HbA1c, urinary ACR, and amount of proteinuria between DN patients with and without cancers (Table 1). Colorectal cancer was the most common cause of cancers in the patients with DN (34.0%), followed by lung (16.4%), liver (13.2%), stomach (6.0%), and breast (5.6%).

The risks of any cancer occurrence were similar between patients with DN and the age-matched general population (SIR 1.05, 95% CI 0.92–1.19). The SIRs of all cancers were then further analyzed according to the sex, age at diagnosis of DN, and year of diagnosis of DN (Table 2). However, there were also no significant differences in cancer risks among male and female patients, age, and calendar year at the diagnosis of DN.

**Table 2:**
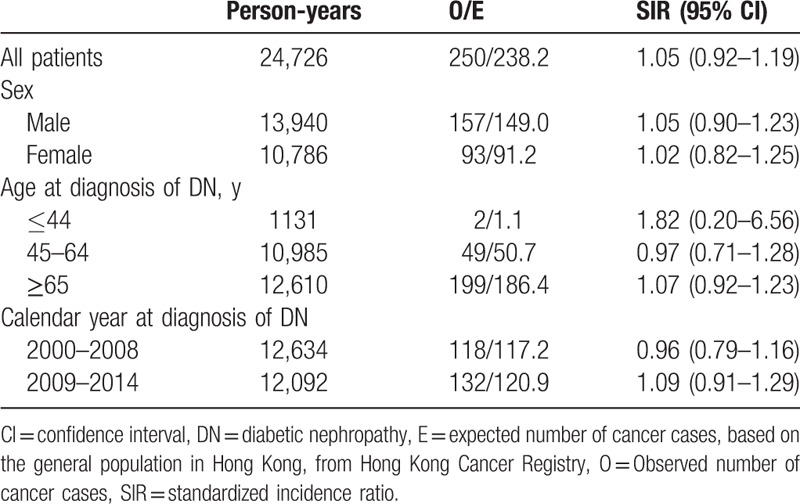
Standardized incidence ratios for all malignancies, stratified by sex, age, and calendar year at diagnosis of DN.

Table 3 showed the SIRs of the major cancer sites. Highest risk was observed for laryngeal cancer (SIR 3.03, 95% CI 1.11–6.60), followed by cancers of liver (SIR 1.96, 95% CI 1.35–2.76) and colorectum (SIR 1.92, 95% CI 1.53–2.37). All patients with laryngeal cancer were males. For cancers of liver and colorectum, the incidence was increased in both male and female patients (Table 4). However, the risk of prostate cancer was lower (SIR 0.48, 95% CI 0.21–0.95) in the males with DN.

**Table 3:**
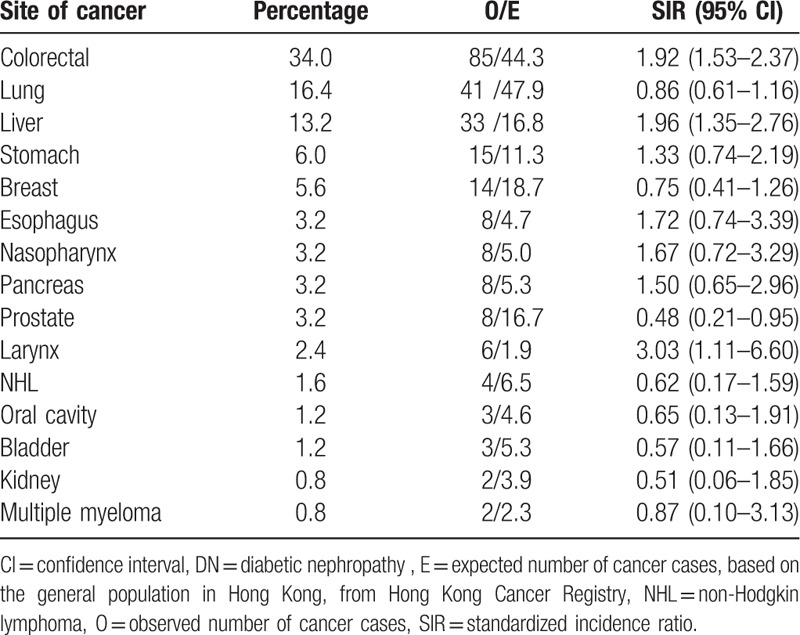
Percentage of different cancer sites and site-specific cancer risk in all patients with DN.

**Table 4:**
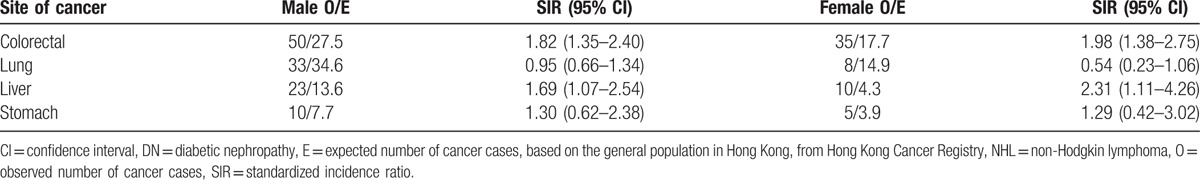
Site-specific cancer risk of commonest cancers in male and female patients with DN.

Total 162 patients died of cancer during the study period. The cancer mortality risk was higher in patients with DN when compared with the general population (SMR 1.17, 95% CI 1.01–1.37). When the SMR of all cancers was further analyzed according to sex, the SMR of male and female patients was 1.18 (95% CI 0.96–1.42) and 1.08 (95% CI 0.82–1.41), respectively. The SMR of common cancer sites were shown in Table 5.

**Table 5:**
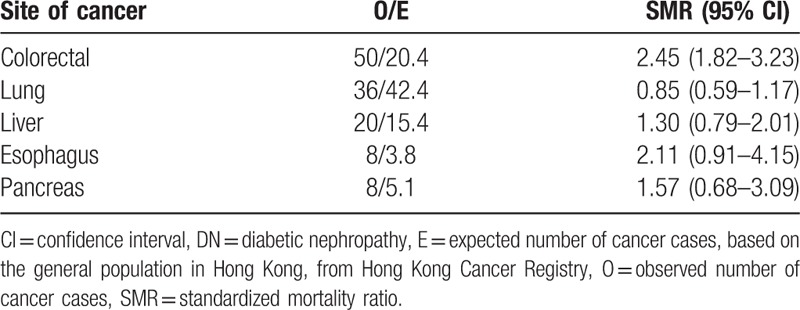
Standardized mortality ratio of the commonest sites of cancer deaths in patients with DN.

## 4 Discussion

The association between DM and the risk of various cancers has been extensively investigated. A number of studies have suggested that DM can increase the risk of several cancers such as pancreatic cancer,^[4]^ liver cancer,^[5]^ colorectal cancer,^[6]^ breast cancer,^[7]^ and kidney cancer,^[8]^ but the exact reasons for the increased risk of these cancers are still a matter of debate. Different mechanisms have been proposed to explain the relationship between DM and risk of cancer. Type 2 DM is associated with relative insulin insufficiency and is characterized by hyperinsulinemia and insulin resistance. Insulin is a growth factor which can promote proliferation of cancer cells. Therefore, hyperinsulinemia can have a direct carcinogenic effect.^[24–26]^ In addition, hyperinsulinemia may also increase the level of insulin-like growth factor axis protein 1, which is a potential mitogen and inhibitor of apoptosis and can promote tumorigenesis.^[27–29]^ Furthermore, type 2 DM and cancers share many common risk factors such as age, obesity, tobacco use, and higher intake of saturated fats. As a result, both insulin resistance and shared risk factors can explain the association between type 2 DM and risk of cancer development. However, type 1 DM is a condition caused by the autoimmune destruction of the beta cells within the pancreas. The evidence of the association between type 1 DM and cancer is limited and variable, depending on the research methods used and the criteria for the diagnosis of DM.^[30]^ In a recent study using one of the largest diabetes registries, the magnitudes of excess risk of cancers for type 1 and type 2 DM are generally similar, supporting the concept that hyperglycemia, found in both types of DM, can be the mechanistic driver between DM and cancers.^[8]^

Despite all the available clinical evidence, there are no data concerning the cancer pattern and the risk of various cancers in patients with DN. The classic renal pathology of DN, no matter whether it is caused by type 1 or type 2 DM, is the presence of different pathological lesions of diabetic glomerulosclerosis. These lesions could then lead to albuminuria. Albuminuria assessment is commonly used for early diagnosis and screening of DN. Microalbuminuria may be regarded as an early marker of DN and can be defined as appearance of small amounts of albumin in urine (30–300 mg/day in 24-hour collection or spot urine ACR 3–30 mg/mmol). However, macroalbuminuria is defined as an ACR >30 mg/mmol. Two of 3 samples should fall within the microalbuminuric or macroalbuminuric range to confirm the classification.^[20]^ Without specific interventions, approximately 80% of patients with type 1 DM who developed sustained microalbuminuria will progress to the stage of macroalbuminuria or overt nephropathy during a period of 10 to 15 years. ESKD develops in 50% of type 1 diabetic patients with overt nephropathy within 10 years and in >75% by 20 years. However, 20% to 40% of type 2 diabetic patients with microalbuminuria progress to overt nephropathy and only 20% will further progress to ESKD by 20 years after onset of overt nephropathy.^[31]^ It has been found that each SD increase in albuminuria could result in a 20% increase in the overall risk of cancer even in patients without DM.^[10]^ Moreover, a study in Taiwan showed that presence of proteinuria could predict 10-year cancer-related mortality in patients with type 2 DM.^[32]^ However, another study based on a cohort from a randomized trial showed that mild to moderate chronic kidney disease (CKD) did not increase the risk of cancer in people with DM.^[33]^

To the best of our knowledge, ours is the first cohort study investigating the risk of various cancers in patients with DM-associated nephropathy when compared with the general population. We could not find any significant association between DN and an increased incidence of overall cancer. However, there were increased risks of certain site-specific cancers such as cancers of larynx, liver, and colorectum in our cohort. Positive association of laryngeal cancer with either DM or CKD was rarely reported in literature. A prospective cohort study in Japan observed a positive association of DM with laryngeal cancer in men.^[34]^ As all the patients with laryngeal cancer in our cohort were males, the SIR of laryngeal cancer in men with DN was 2.94 (95% CI 1.07–6.40). As the number of patients is small and confounding factors such as smoking status are not known, further studies are required to ascertain this positive association.

In addition, our findings also indicated that patients with DN had a higher incidence of liver cancer (SIR 1.96, 95% CI 1.35–2.76) and colorectal cancer (SIR 1.92, 95% CI 1.53–2.37) when compared with the general population. In fact, the positive association between both cancers and DM has been well documented in literature.^[5,6]^ In a meta-analysis, the incidence of liver cancer was higher in individuals with DM (SIRs = 2.01, 95% CI: 1.61–2.51), compared with those without DM.^[5]^ Moreover, DM was associated with an increased risk of colorectal cancer, compared with no DM (SIR 1.30, 95% CI 1.20–1.40).^[6]^ However, we observed a lower risk of prostate cancer in our cohort. This is in concordance with some studies in patients with DM^[8]^ and the reason can be owing to the lower levels of circulating testosterone in these individuals.^[35]^ Unlike some other studies, we did not find any significant association between DN and cancers of pancreas, breast, or kidneys.

The overall risk of cancer death in patients with DN was significantly higher than the age- and sex-matched population in our study. However, among the different cancers identified in our cohort, only colorectal cancer had higher cancer mortality than the general population (SMR 2.45, 95% CI 1.82–3.23). Proteinuria has been found to be associated with cancer-related mortality (hazard ratio 1.99; 95% CI 1.00–3.94). In addition, proteinuria also showed a trend of increased colon cancer death.^[30]^ The exact mechanisms for the association between proteinuria and cancer mortality remain unclear. One possible explanation is that proteinuria is associated with low-grade inflammation in DM, which can increase the risk of cancer.^[36]^

There are several strengths in this study. First of all, our study has the largest patient-years of follow up in studying the relationship between DN and cancers. Moreover, an accurate ascertainment of those patients with DN and cancers was possible by linking the various data in the CDARS. The database generated from CDARS has been used to conduct high-quality epidemiological studies in the past.^[37,38]^ In addition, the cancer data obtained from the Hong Kong Cancer Registry are also of high standard. The percentage of histologically verified cases and death certificate only cases (the 2 key performance indicators of data completeness) was 87% and 0.7%, respectively. This is already up to the highest International Association of Cancer Registries standard for developed countries ^[23]^

However, there are a number of limitations in our study. First of all, it was a retrospective study and various risk factors of cancers such as obesity, tobacco use, alcohol consumption, and family history of cancers were not recorded. In addition, DM itself is not a single disease, but can be viewed as a group of metabolic disorders characterized by hyperglycemia. A series of potential confounders directly related to DM such as obesity, diet, and physical activity were not available in our study. Moreover, the diagnosis of DN might be underestimated in our study because it was based on the presence of albuminuria only. Although the natural history of DN has been described as progressive albuminuria followed by a steady loss of GFR, there are studies showing that patients with DN can have a reduced GFR without albuminuria.^[39]^ In our study, presence of albuminuria was assessed using a single specimen. As albuminuria can be transient or reversible, only those patients with macroalbuminuria and/or proteinuria were included. It has been known that transient hyperglycemia, urinary tract infections, exercise within 24 hours, severe hypertension, congestive heart failure, and acute febrile illness can all cause transient microalbuminuria. Some studies have shown that although all individuals with macroalbuminuria in the first urine sample had either macroalbuminuria or microalbuminuria in the second urine sample, approximately one-third of the adults with microalbuminuria would not have increased urine albumin excretion on repeat assessment.^[39,40]^ Furthermore, we might also miss some patients with DN who had improvement of albuminuria or proteinuria after the use of angiotensin-converting enzyme inhibitors or angiotensin receptor blockers. As we only focused on those patients with DN, the size of our cohort was relatively small and there was insufficient power to provide sex-specific estimates of each cancer from this study. In addition, the findings in our study might not be able to be generalized to other populations because the cancer risk was different among diabetic patients of different ethnicities.^[41]^ Finally, the information concerning the use of antidiabetic medication such as metformin, which has been found to be associated with reduced risk of cancers in some studies, were not included in our analysis.^[42,43]^

In conclusion, we demonstrate that DN is associated with increased incidence of cancers of colorectum, liver, and larynx but decreased incidence of prostate cancer. Moreover, there is also increased mortality of colorectal cancer in patients with DN. As most of the patients in this study are Chinese, further studies are required to see whether similar association can also be found in other populations.

Our manuscript is a unique submission and is not being considered for publication by any other source in any medium. Further, the manuscript has not been published, in part or in full, in any form.
